# An in vivo study to investigate an original intramedullary bone graft harvesting technology

**DOI:** 10.1186/s40001-023-01328-8

**Published:** 2023-09-15

**Authors:** Markus Laubach, Agathe Bessot, Jacqui McGovern, Siamak Saifzadeh, Jonathan Gospos, Daniel N. Segina, Philipp Kobbe, Frank Hildebrand, Marie-Luise Wille, Nathalie Bock, Dietmar W. Hutmacher

**Affiliations:** 1 Australian Research Council (ARC) Training Centre for Multiscale 3D Imaging, Modelling, and Manufacturing (M3D Innovation), Queensland University of Technology, Brisbane, QLD 4000 Australia; 2 Centre for Biomedical Technologies, School of Mechanical, Medical and Process Engineering, Queensland University of Technology, Brisbane, QLD 4059 Australia; 3 Department of Orthopaedics, Trauma and Reconstructive Surgery, RWTH Aachen University Hospital, Pauwelsstraße 30, 52074 Aachen, Germany; 4 Max Planck Queensland Centre (MPQC) for the Materials Science of Extracellular Matrices, Queensland University of Technology, Brisbane, QLD 4000 Australia; 5 Centre for Biomedical Technologies, School of Biomedical Sciences, Faculty of Health, and Translational Research Institute (TRI), Queensland University of Technology (QUT), Brisbane, QLD 4102 Australia; 6 ARC Training Centre for Cell and Tissue Engineering Technologies, Queensland University of Technology (QUT), Brisbane, QLD 4000 Australia; 7 Medical Engineering Research Facility, Queensland University of Technology, Chermside, QLD 4032 Australia; 8 Department of Orthopaedics, Holmes Regional Trauma Center, Melbourne, FL USA; 9 Department of Trauma and Reconstructive Surgery, BG Klinikum Bergmannstrost, Halle, Germany; 10 Department of Trauma and Reconstructive Surgery, University Hospital Halle, Halle, Germany

**Keywords:** Bone graft, Harvesting, Growth factors, Cytokines

## Abstract

**Background:**

Harvesting bone graft (BG) from the intramedullary canal to treat bone defects is largely conducted using the Reamer–Irrigator–Aspirator (RIA) system. The RIA system uses irrigation fluid during harvesting, which may result in washout of osteoinductive factors. Here, we propose a new harvesting technology dedicated to improving BG collection without the potential washout effect of osteoinductive factors associated with irrigation fluid. This novel technology involves the conceptual approach of first aspirating the bone marrow (BM) with a novel aspirator prototype, followed by reaming with standard reamers and collecting the bone chips with the aspirator (reaming–aspiration method, R–A method). The aim of this study was to assess the harvesting efficacy and osteoinductive profile of the BG harvested with RIA 2 system (RIA 2 group) compared to the novel harvesting concept (aspirator + R–A method, ARA group).

**Methods:**

Pre-planning computed tomography (CT) imaging was conducted on 16 sheep to determine the femoral isthmus canal diameter. In this non-recovery study, sheep were divided into two groups: RIA 2 group (*n* = 8) and ARA group (*n* = 8). We measured BG weight collected from left femur and determined femoral cortical bone volume reduction in postoperative CT imaging. Growth factor and inflammatory cytokine amounts of the BGs were quantified using enzyme-linked immunosorbent assay (ELISA) methods.

**Results:**

The use of the stand-alone novel aspirator in BM collection, and in harvesting BG when the aspirator is used in conjunction with sequential reaming (R–A method) was proven feasible. ELISA results showed that the collected BG contained relevant amounts of growth factors and inflammatory cytokines in both the RIA 2 and the ARA group.

**Conclusions:**

Here, we present the first results of an innovative concept for harvesting intramedullary BG. It is a prototype of a novel aspirator technology that enables the stepwise harvesting of first BM and subsequent bone chips from the intramedullary canal of long bones. Both the BG collected with the RIA 2 system and the aspirator prototype had the capacity to preserve the BG’s osteoinductive microenvironment. Future in vivo studies are required to confirm the bone regenerative capacity of BG harvested with the innovative harvesting technology.

**Supplementary Information:**

The online version contains supplementary material available at 10.1186/s40001-023-01328-8.

## Introduction

Bone graft (BG) collected from the medullary canal of lower leg long bones is well-recognized for its bone regenerative capacity [1]. For historical and biological reasons, autologous BG is considered the gold standard among graft materials [2]. Indeed, autologous BG is the only graft material that fulfils all three components of the regeneration triad, namely, being of high osteogenic, osteoinductive, and osteoconductive capacity [3]. It is transplanted 2.2 million times annually worldwide [4], which makes it the most transplanted tissue after blood [5, 6]. The iliac crest (IC) has traditionally been considered the gold standard source; however, this harvesting site is limited by several factors, including limited BG volume, donor site morbidity with persistent pain at the IC in up to 30% of cases [7, 8], and a limited amount of available and biologically active cells in the graft [9], with particularly fewer stem cells after the age of 55 [10]. Due to the limitations associated with harvesting BG from the IC, identifying alternative harvest sites has become a necessity. Collection of adequate volumes of BG from the femur or tibia can be performed by applying continuous irrigation and simultaneous aspiration of the irrigation fluid, using a device introduced by Synthes called the Reamer–Irrigator–Aspirator (RIA) system (RIA 1 system, 2005 version) [11]. The RIA system allows for BG to be harvested from the femur with fewer complications in comparison with IC [12]. It has been shown that the BG mixture of bone marrow (BM) and bone chips obtainable with the RIA system has osteogenic, osteoinductive, and osteoconductive properties, which have been successfully used especially in the treatment of (large segmental) bone defects [13–16]. In 2019, a second-generation RIA system (RIA 2 system) was released, which includes a smaller diameter of the reamer head (starting at 10 mm compared to 12 mm in RIA 1 system) [17].

Several decades ago, BGs obtained from the reaming debris of long bones were identified as a source of osteogenic cells and osteoinductive signaling proteins, such as growth factors (GF) and cytokines [1, 18, 19]. In particular, the extracellular matrix (ECM) of BG contains specific signaling proteins at a physiological dose and in a ‘non-recombinant’ state [20] resulting in its high capacity to regenerate bone defects [2]. These signaling proteins that enhance bone healing include GFs such as vascular endothelial growth factor (VEGF) [21], insulin-like growth factor (IGF) [22], transforming growth factor (TGF)‑β [22], fibroblast growth factor (FGF) [23, 24], and bone morphogenetic proteins (BMP) [23, 25].

Furthermore, Bolander [26] proposed that a cascade of cellular events crucial for bone healing is triggered by macrophage-derived signaling molecules, which are key regulators of cellular proliferation, differentiation, and ECM synthesis. Macrophages are essential effectors of the innate immune system and can be divided into inflammatory (circulating) and tissue-resident macrophages [27]. Bone tissue-resident macrophages can be divided into BM macrophages, including erythroblastic island macrophages, hematopoietic stem cell niche macrophages, and osteal macrophages, which are also named osteomacs [28]. Thus, in addition to GFs, pro- and anti-inflammatory cytokines, mainly derived from macrophages, are distinctive bone regeneration-related factors [29], and their relevance for bone healing is captured by the term osteoimmunology. Moreover, bone healing begins immediately after the injury in the inflammatory phase when GFs from platelets and macrophage-derived inflammatory factors are released into the hematoma [26] after blood vessel rupture inside bone and surrounding soft tissue, producing a fibrin scaffold that is critical to bone healing [29, 30]. Furthermore, osteomacs are found in proximity to bone lining cells (osteoblasts, osteoclasts), for example, on the endosteum [31], which have the capacity to induce osteoblast differentiation in vivo [32, 33] and facilitation of bone formation [34]. Distinct functional abilities are acquired by macrophages (both resident and inflammatory macrophages) with different phenotypes. Polarized macrophages include pro-inflammatory M1 (classically activated macrophages) and anti-inflammatory M2 (alternatively activated macrophages) induced by microenvironmental stimuli [35]. After activation, M1 macrophages predominantly secrete pro-inflammatory cytokines, such as interleukin (IL)-1β, IL-6 and IL-8, whereas M2 macrophages produce anti-inflammatory cytokines, such as IL-10 [36–40]. Initial inflammatory processes in the hematoma are the starting point initiating the healing cascade [29] and, thereby, are crucial in determining bone healing outcome [41–43].

The effects of different BG harvesting techniques on GF quantities have been described [44, 45]. Moreover, “waste-water” produced during RIA system application (RIA filtrate) contains mesenchymal stem cells (MSC) and osteoinductive proteins that could potentially promote bone healing [46, 47]. MSCs collected from RIA waste-water are as viable as BM cells from IC and present in greater numbers, while the cell types are comparable in terms of osteogenicity [48]. Moreover, the traditionally discarded fatty waste of RIA filtrate may have bone-forming capabilities [49]. However, re-use of RIA filtrate is problematic, because the amount of MSCs and osteoinductive proteins per unit volume is limited due to high dilution during the BG harvesting procedure [50]. Therefore, a promising strategy in regenerative therapy would seem to be harvesting BG from the intramedullary canal of long bones without the potential washout effect associated with the RIA system.

More recently, orthopaedic researchers have also focused their attention on the BM for musculoskeletal regeneration, particularly as a potential clinical therapeutic tool [51]. The BM contains progenitor cells, together with GFs and inflammatory cytokines [52]. The ability of transplanting fresh BM by transferring all the regenerative potential present in the BM environment to the lesion site allows the graft harvest to be processed directly in the operating theatre, which had already been developed about 20 years ago [53]. For instance, the application of BM with or without additional biomaterials has proven to be effective in preclinical studies for treating bone defects [54] as well as a wide range of orthopaedic surgeries, including spinal fusion [55, 56] and tibial fracture healing [57, 58]. The most relevant site for BM aspiration is the IC. However, BM has only a small percentage of the total cell pool [59] and the quality of the aspirate is highly technique dependent and contingent upon the volume of BM aspirated, as there is significant dilution of the aspirate with peripheral blood when the aspirate collection exceeds 4–5 mL [60–62]. Therefore, to routinely achieve a standardized method of aspirating BM in larger volumes than at the IC and without aspirate dilution, an innovative aspiration device is required to allow access to alternative harvesting sites.

**Fig. 1:**
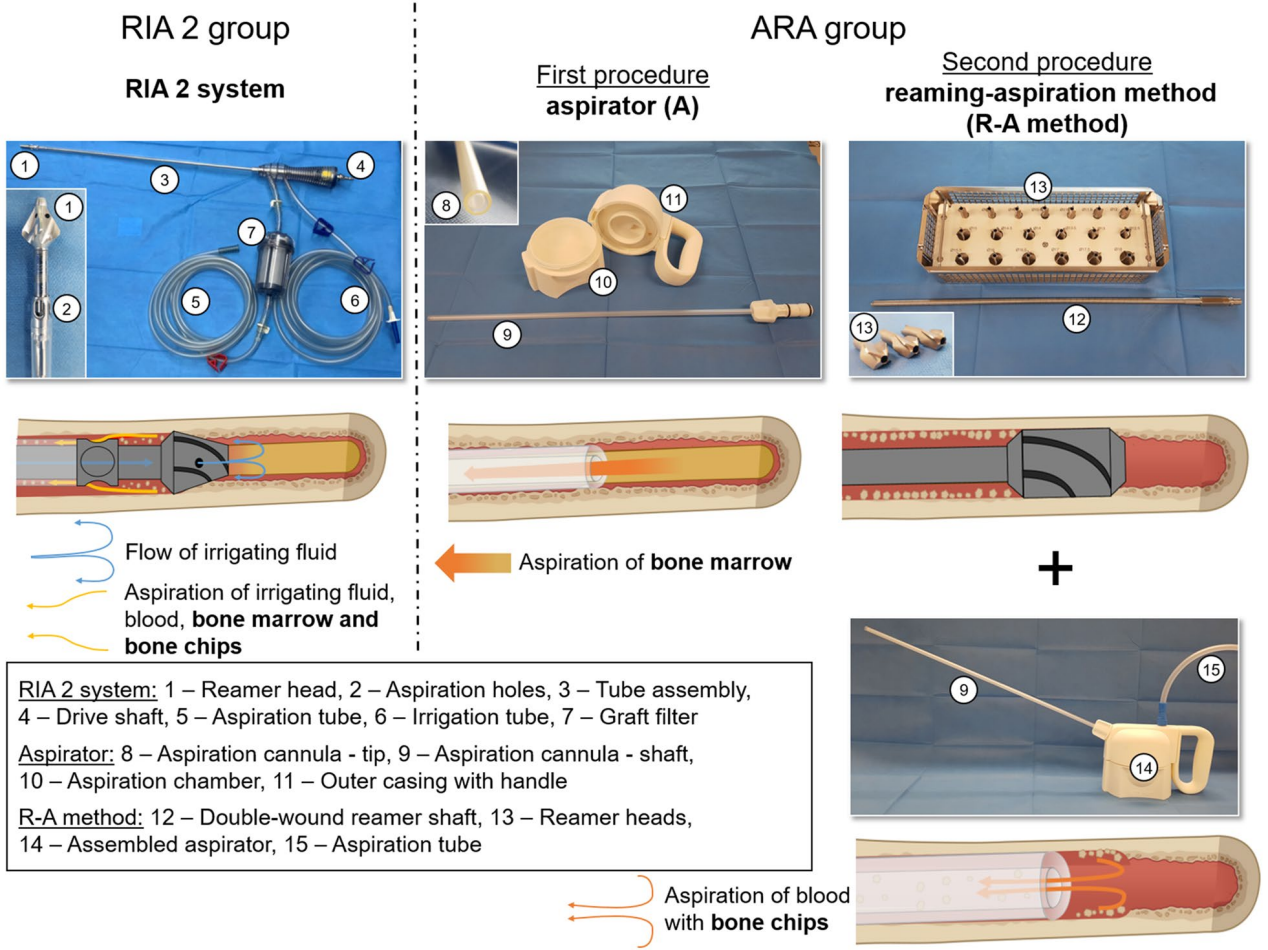
Depiction of the RIA 2 system for harvesting intramedullary BG (mixture of BM and bone chips) and the novel aspirator prototype for BM harvesting as well as for the R–A method (harvesting bone chips). The RIA 2 system is comprised of a reamer head connected to a drive shaft, which is located in a tube assembly. This construct is connected to an aspiration port, which is connected to the operating room wall vacuum over a graft filter to capture the BG. BG harvesting with the RIA 2 device is a one-step procedure with continuous flow of irrigation fluid during reaming, which allows for evacuation of diluted BM and bone chips. The novel aspirator prototype allows for harvesting BM after opening the bone by intruding the intramedullary canal with a specific cannula under continuous suction. Optionally, after emptying the medullary canal of BM, bone chips can be harvested applying iterative intramedullary reaming and aspiration (R–A method). Partially created with BioRender.com

Henceforth, a new aspiration and BG collection device was conceptualized. The novel aspirator allows for harvesting BM utilizing standard surgical access to long bones. In addition, a two-step method of intramedullary “reaming” using a standard reamer kit and “aspiration” with the aspirator device (reaming–aspiration method, R–A method) allows for harvesting of bone chips. In contrast to the conventional one-step RIA 2 system of BG harvesting, the novel aspirator operates without continuous reaming and irrigation (Fig. 1). Here, we evaluated the harvesting efficacy and osteoinductive profile of BG harvested with the innovative intramedullary harvesting concept (aspirator + R–A method, ARA group), which allows separate harvesting of BM (aspirator) and bone chips (R–A method) through the novel technology used, and compared it with the BG mixture of BM and bone chips harvested with the clinically routine RIA 2 system (RIA 2 group).

## Materials and methods

### Animal ethics approval, code of practice, and ARRIVE 2.0 guidelines

Ethical approval was obtained from the Queensland University of Technology (QUT) Animal Ethics Committee (UAEC) (Ethics Approval Number 2000000593). All cadaveric and in vivo work were performed at the QUT Medical Engineering Research Facility (MERF) at Prince Charles Hospital campus (Chermside, Queensland, Australia). The sheep (Ovis aries, *n* = 16, breed: Merino, sex: female, age: 1–2 years, body weight: 41–51 kg) were procured from a local farm and preoperative health check performed by a veterinarian as per a previously validated protocol [63]. The study was conducted in accordance with the requirements of the Australian Code of Practice for the Care and Use of Animals for Scientific Purposes and the ARRIVE 2.0 guidelines (Animal Research: Reporting of In Vivo Experiments) [64] were followed.

### Overview of the novel aspirator device

The prototype of a novel aspirator device was designed, manufactured, and provided by Stryker. It was three-dimensional printed by applying the additive manufacturing technique of fused deposition modeling using fine powder polyamide (PA) polymer 2200 based on PA 12 (also known as Nylon 12). Subsequently, the individual devices, including the aspiration cannulas, were double-packed and sterilized with gamma irradiation. This device comprises three main components, namely, a modular aspiration chamber, a flexible entry-aspiration elongated port (cannula) to provide (intramedullary) access and collect BG, and an outer casing with a handle equipped with a dedicated suction outlet (Fig. 1).

### Preoperative femoral computed tomography imaging

Under brief general anesthesia using isoflurane, all sheep were placed on the computed tomography (CT) table in a head-first supine position and subjected to preplanning femoral CT imaging. A high-resolution helical acquisition was completed using a single-source CT (Toshiba Aquilion Lightning^™^, Tokyo, Japan). Scanning was performed in a craniocaudal direction from the IC to the proximal tibia. Soft tissue and sharp bone axial data sets were reconstructed with a section thickness of 1.0 mm (increment, 0.5 mm). All data sets were exported as a digital imaging and communications in medicine (DICOM) file for further analysis.

### Allocation of animals to experimental groups

Preoperative CT data (DICOMs) were imported in the open-source medical image viewer Horos (version 3.3.6). Using this software, the smallest diameter (isthmus) was determined by first measuring the length of the femur, as defined from the trochanteric fossa to the condyles. Subsequently, the intramedullary canal diameter was determined at the midway point of the total femur length. Representative images to illustrate the calculations are provided in Additional file 2: Fig. S1. The allocation of sheep into the experimental groups for the RIA 2 system (RIA 2 group) and the aspirator + R–A method (ARA group) was based on the smallest medullary diameter of the femoral shaft, with the objective of achieving matching of sheep in both groups with similar intramedullary diameters.

### Surgical procedure and bone graft collection

Prior to the commencement of the in vivo study, a series of cadaveric studies were performed to acquire a thorough understanding of the anatomy to anticipate and to avoid potential complications (Additional file 2: Fig. S2). In the in vivo study, 16 fully anesthetized sheep with suitable pain relief were assigned to the RIA 2 group (*n* = 8) and the ARA group (*n* = 8). They were positioned in right lateral recumbency, and surgical skin preparation was performed as previously described [63] while ensuring sufficient accessibility to the hip joint for anterograde approach of the left proximal femur. All surgical interventions were performed by the same surgeon (M.L.) under the same surgical setup (Additional file 2: Fig. S3), and surgical duration was determined from skin incision to provisional stapler closure after the last BG harvesting step. For surgical access, the femur was angled at 90° in the hip joint, the greater trochanter palpated, and a longitudinal skin incision was made 1–2 cm proximal to the greater trochanter. Subsequently, the biceps femoris muscle was incised, and the tendon of iliacus muscle was identified and severed. The overlying tissue on the trochanteric fossa was scraped off with a periosteal elevator. Under X-ray surveillance, a K-wire was introduced, a cannulated rigid reamer Ø10 mm (Stryker) was inserted over the K-wire to access the femoral medullary canal via the trochanteric fossa, followed by placement of a ball tip guide wire. The femoral opening was then enlarged with an Ø11 mm BixCut fixed-head reamer (Stryker). Additional file 2: Fig. S4 depicts the animal positioning and surgical approach to the femur.

Harvesting of BG was performed with either the RIA 2 system (RIA 2 group) or the aspirator prototype, followed by the R–A method (ARA group). The RIA 2 system is an assembly of an irrigation and aspiration setup in which the reaming to the desired diameter per reamer head size is done in a single step. The RIA 2 system was applied starting with a reamer head 2 mm narrower than the preoperatively measured diameter of the femoral isthmus with increments of 1 mm reamer head size for the first two reaming steps. Subsequently, the reamer head size was increased by 0.5 mm steps for iterative reaming until approximately 0.5–1 mm of residual cortex at the isthmus was observed on conventional X-ray imaging. In the ARA group, BG deposited on the reamer head was manually collected, while in the RIA 2 group, the harvested BG, including bone chips, was aspirated from the RIA 2 system reamer head, leaving this step superfluous. In the ARA group, during the first step (= aspiration step), BM was aspirated using the aspirator prototype. During the second step (= R–A method), reaming was performed using the BixCut reamer, in sequential steps with increments described above for the RIA 2 group. Following each reaming step, to collect morselized endosteal bone particles (bone chips), the aspirator’s nozzle was introduced into the medullary canal in a reciprocating (back-and-forth) fashion, under repetitive advancing and retracting movement with gently touching the endocortex. All animals were humanely killed on the day of surgery without recovery from anesthesia with 100 mg/kg body weight pentobarbital sodium (Lethabarb^®^) intravenously.

Irrespective of the BG collecting method, the harvested graft was removed from the canisters, and weighed as three different graft groups: (1) RIA 2 system; (2) aspirator; and (3) R–A method. The net weight of the collected BG was determined after syringe aspiration of the aqueous BG components. The aqueous BG components collected resulted in two additional groups: RIA 2 system—aqueous BG component and R–A method—aqueous BG component. The aspirator BM group did not yield relevant volumes of aqueous BG components.

### Analyses of CT imaging for the assessment of femoral cortex reduction

Following euthanasia, additional ex vivo CT scans of the femora were performed based on the protocol described in the *Preoperative femoral computed tomography imaging* section to compare pre- and postoperative cortical bone volume. The segmentation of the CT data of the femora was performed using Mimics software (Mimics v20.0, Materialise, Leuven, Belgium) and conducted with the upper threshold of the cortical bone of 1200 Hounsfield Units (HU). The threshold of cortical bone to spongiosa was set to 580 HU. The outer and inner shapes of the cortical bone were segmented by applying these thresholds. The cortical wall and associated cortical thickness (bone volume) were derived by subtracting the cancellous bone shape from the cortical shape. The cortical volume reduction after the reaming process was analyzed using Geomagic software (Geomagic Control 2014.3.0, 3D Systems, Rock Hill, USA). Overall, the main bone volume reduction was observed for the middle 40% of the femoral bone length; therefore, bone volume reduction was calculated in this section. Representative images that illustrate the process of bone volume reduction calculations are provided in Additional file 2: Fig. S5.

### Processing and analyzing harvested bone graft

#### Sample preparation

To extract proteins, fresh BG was digested in a standardized volume of Dulbecco’s Modified Eagle Medium (Thermo Fisher, cat# 11960069) containing 1% penicillin/streptomycin (Thermo Fisher, cat# 15140122) and collagenase type II (270 U/mL; Thermo Fisher, cat# 17,101,015) equivalent to the weight of the BG (g/mL) during consistent motion in a shaking incubator (200 rpm) at 37 °C for 90 min. Subsequently, the samples were filtered with 70 μm cell strainer (Falcon, cat# 352350), the filtrate was centrifuged (1000 g for 20 min), and the supernatant was collected and frozen (− 80 °C) until further analysis.

After aspiration of RIA 2 system and R–A method BG fluids (aqueous BG components), these aspirates were transferred to EDTA blood tubes (Sarstedt, cat# 01.1605.100) (Additional file 1: Video S1) and subjected to stepwise centrifugation to remove cellular debris. The samples were first centrifuged at 3000 g for 15 min at 4 °C. The supernatant was aspirated and centrifuged again at 3000 g for 15 min at 4 °C, and then collected and frozen (-80 °C).

#### Analyses of growth factor, total protein, and inflammatory cytokine concentrations

The amount of GFs and inflammatory cytokines in the samples were quantified by enzyme-linked immunosorbent assay (ELISA) methods and, in the case of the hard tissue components, normalized to the weight of tissue analyzed. The amount of the following GFs was determined: VEGF (cat# MBS1602118), IGF-1 (cat# MBS2510826), TGF-β1 (cat# MBS1602080), ‘basic’ FGF-2 (cat# MBS734168), BMP-2 (cat# MBS2512267), and BMP-4 (cat# MBS012205). All ELISA kits were sheep specific and were obtained from MyBioSource, Inc. The kits were used in accordance with the manufacturer’s instructions, with standards and samples in duplicate. The sensitivity for each respective ELISA is as follows: VEGF (2.46 ng/L), IGF-1 (4.688 ng/mL), TGF-β1 (0.022 ng/mL), FGF-2 (1.0 pg/mL), BMP-2 (18.75 pg/mL), and BMP-4 (1.0 pg/mL). Total protein within the samples was quantified using a bicinchoninic acid (BCA) colorimetric assay (Pierce^™^ BCA Protein Assay Kit, Thermo Fisher, cat# 23,225) with standards and samples in triplicate. Furthermore, as per the protocol of Bouquet et al. [65], standard sandwich ELISAs for the inflammatory cytokines of IL-1β, IL-6, IL-8 and IL-10 were conducted with standards and samples in duplicate. Recombinant and antibody pairing details and additional reagent details for the ELISAs of the inflammatory cytokines (IL-1β, IL-6, IL-8, IL-10) are provided in Additional file 2: Table S1 and for final reagent working concentrations we refer to the protocol of Bouquet et al. [65]. Sensitivities of IL-1β, IL-6, IL-8 and IL-10 were given as 117.6 pg/mL, 443.1 pg/mL, 30.9 pg/mL, and 64.3 pg/mL, respectively [65]. Except for the sample and standard dispensing and plate washing steps, a pipetting robot (epMotion^®^ 5073 M, software version 40.5.3.1) was used for the IL-1β, IL-6, IL-8, and IL-10 complex ELISA protocols to increase reliability and reproducibility in the interest of improved standardization [66]. The absorbance of all ELISA plates was read at 450 nm. A standard curve was created, and a four-parameter logistic (sigmoidal, 4-PL) curve fit was used to determine the sample and standard concentrations (GraphPad Prism 9.3.0, CA, USA).

### Statistical analysis

As this study was explorative, in line with previous suggestions, we focused on descriptive statistics [67]. Thus, future studies are required for independent, statistically rigorous confirmation, including assessing statistical significances between treatment groups [68]. Nonetheless, all results are presented either with mean and standard deviation ( ±) or as a boxplot, always describing the observed trends. However, no definite conclusions regarding statistical significance were made.

## Results

**Fig. 2:**
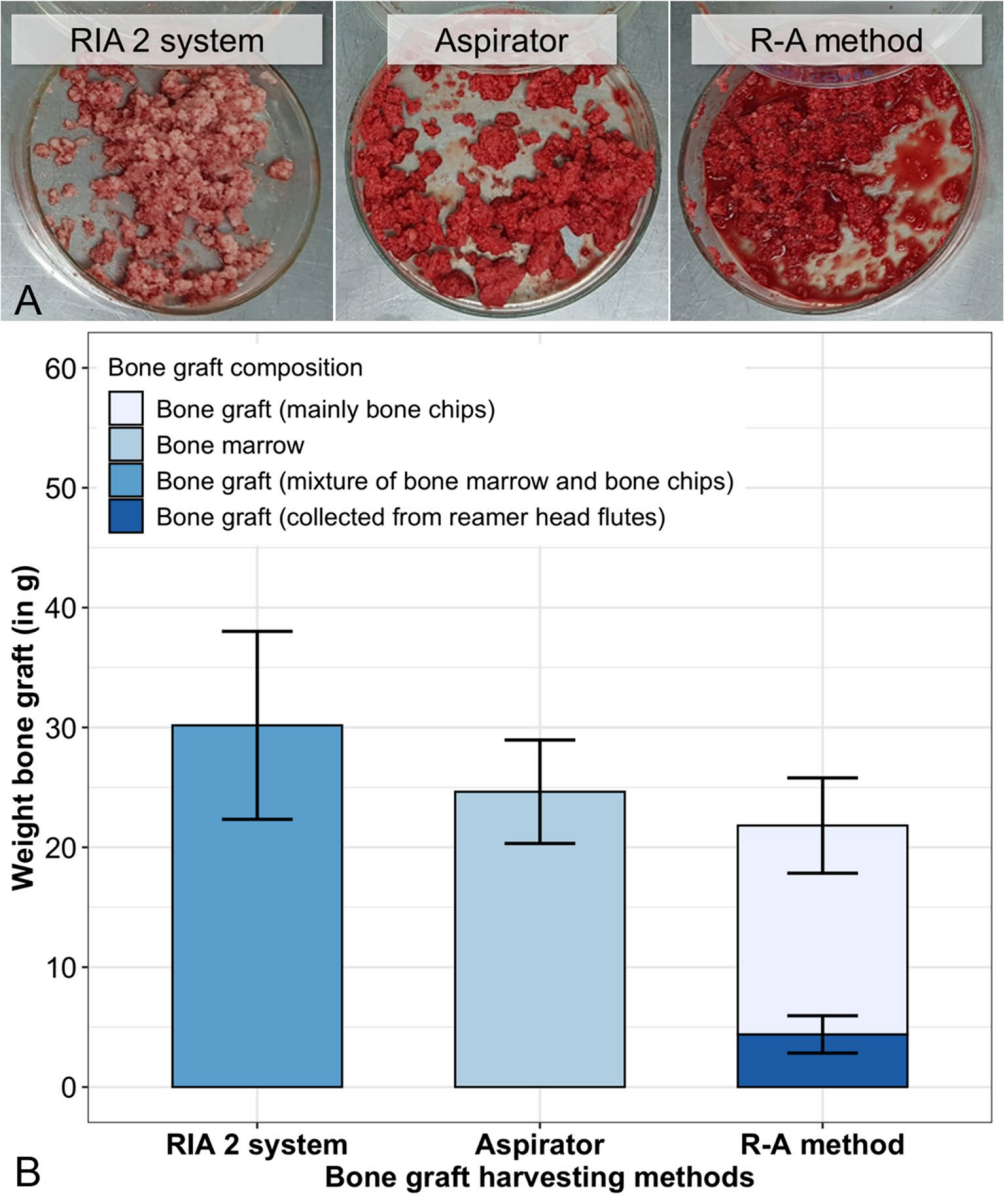
Representative images of the BG and bar chart with the determined weight of the harvested BG. Illustrative pictures of the harvested BG after aspiration of the aqueous BG components **A**. Weight of the harvested BG **B**. Means ± SD, n = 8

**Fig. 3:**
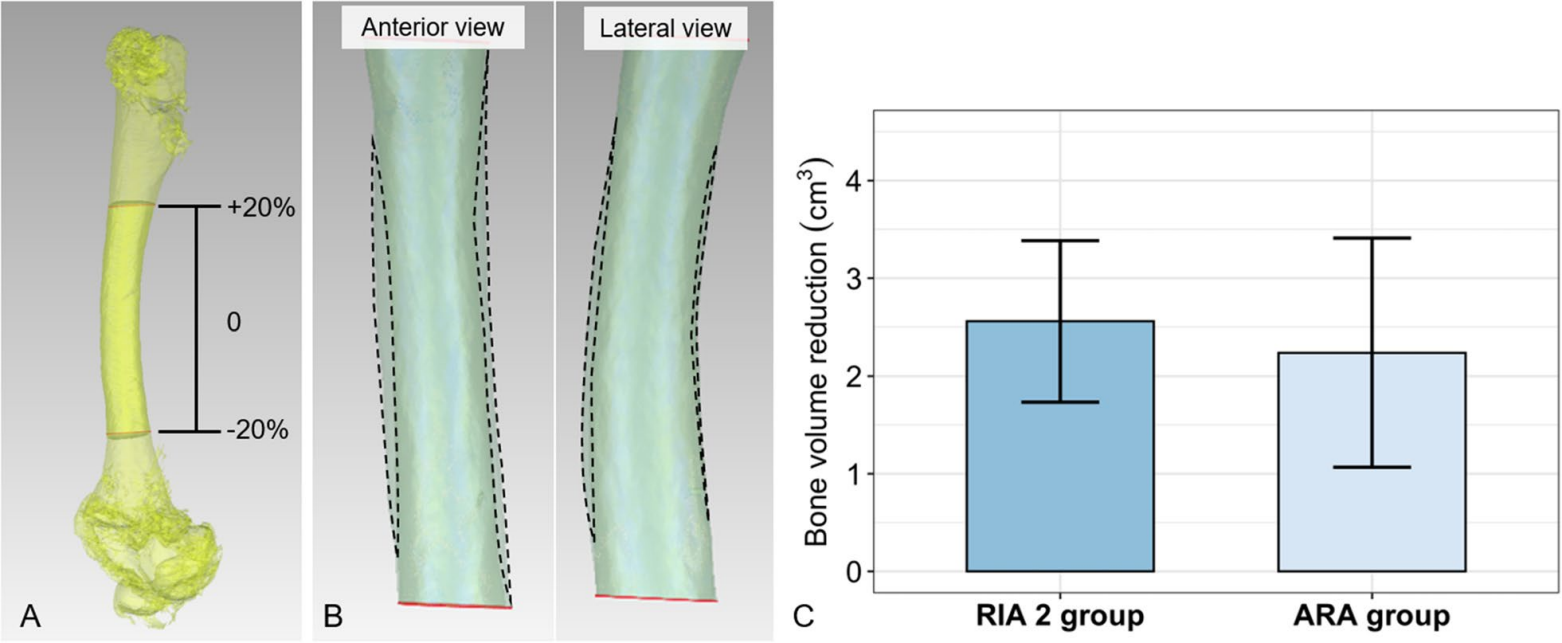
Reduction of femoral cortical bone volume after BG harvesting based on a comparison of preoperative and postoperative CT scans. Reaming volume was determined to be between + 20% and − 20% from the middle of the bone **A**. Representative images of cylinder volume, i.e., volume difference due to reaming, indicated by dashed lines, measured as increase in intramedullary volume in pre- and postoperative comparison **B**. Similar to slightly higher bone volume reduction for the RIA 2 compared to the ARA group **C**. Means ± SD, *n* = 8

Based on preoperative CT scanning, sheep were assigned to two groups: the RIA 2 group and the ARA group, with similar narrowest mean medullary canal diameters measuring 12.21 ± 0.37 mm and 12.29 ± 0.41 mm, respectively. In total, 73 reaming sequential steps were performed in the RIA 2 group, with a mean volume of irrigation of isotonic 0.9% NaCl of 1525 ± 102 mL per surgery, and 74 reaming sequential steps in the ARA group. The mean duration of surgery tended to be shorter in the RIA 2 group (46.8 ± 8.3 min) compared to the ARA group (54.1 ± 7.3 min). In the RIA 2 group, the mean weight of BG was 30.2 ± 7.8 g and the mean weight of the BM collected using the aspirator prototype was 24.6 ± 4.3 g. The BG harvested applying the R–A method was composed of BG deposits from the reamer head (4.4 ± 1.6 g) and BG from sequential reaming and aspiration (17.4 ± 4.0 g) (Fig. 2). Postoperative versus preoperative CT images demonstrated a cortical bone volume reduction for the RIA 2 and the ARA group of 2.56 ± 0.83 cm^3^ and of 2.24 ± 1.17 cm^3^, respectively (Fig. 3). Therefore, harvesting with both the RIA 2 and the ARA group resulted in similar cortical bone volume reduction.

**Fig. 4:**
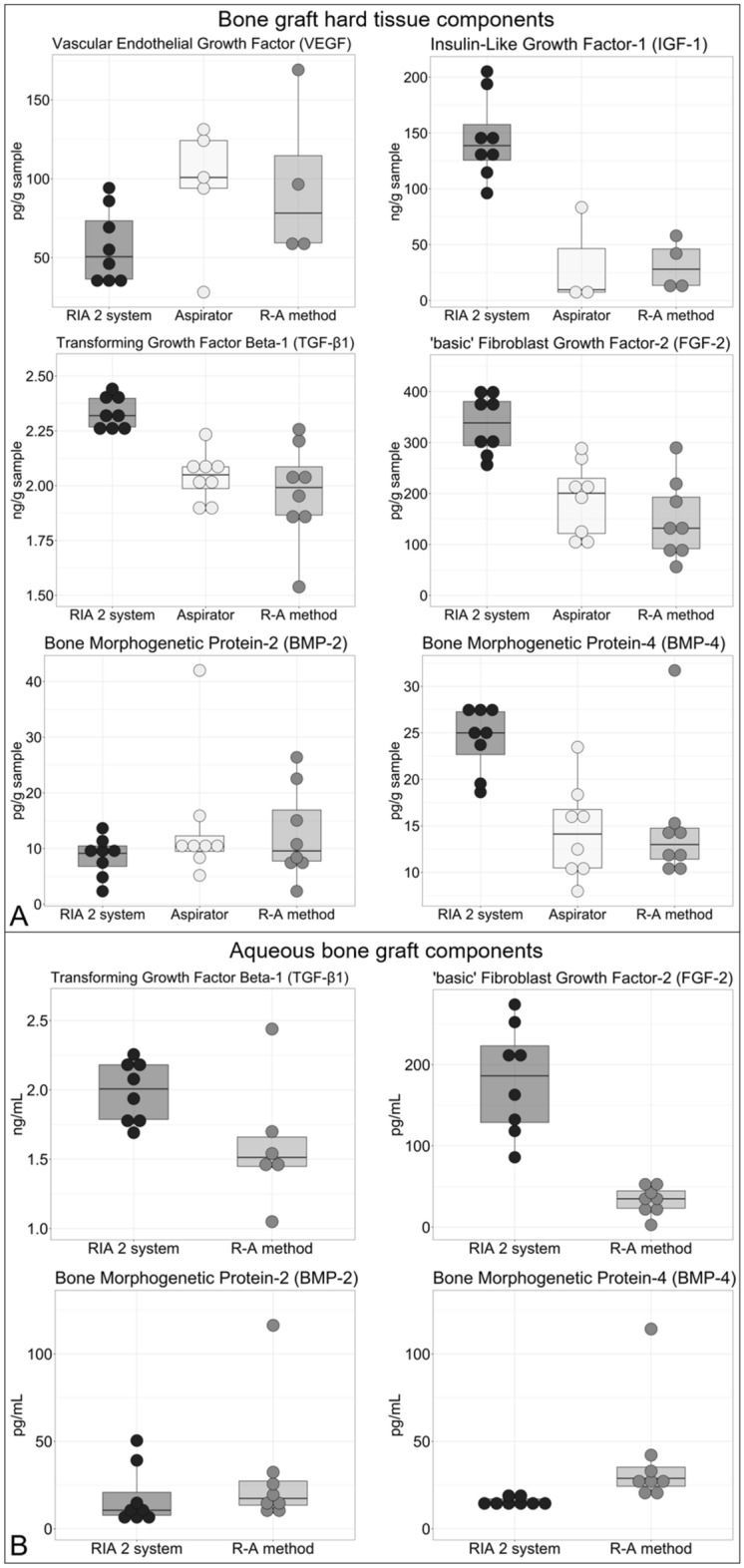
Boxplots of BG growth factor amount by harvesting method. Relevant amounts of GFs were detected in BG hard tissue components **A** and aqueous BG components **B** in all three harvesting methods. Please note that if less than eight dots per group are shown, missing measurements were below the detection range

**Fig. 5:**
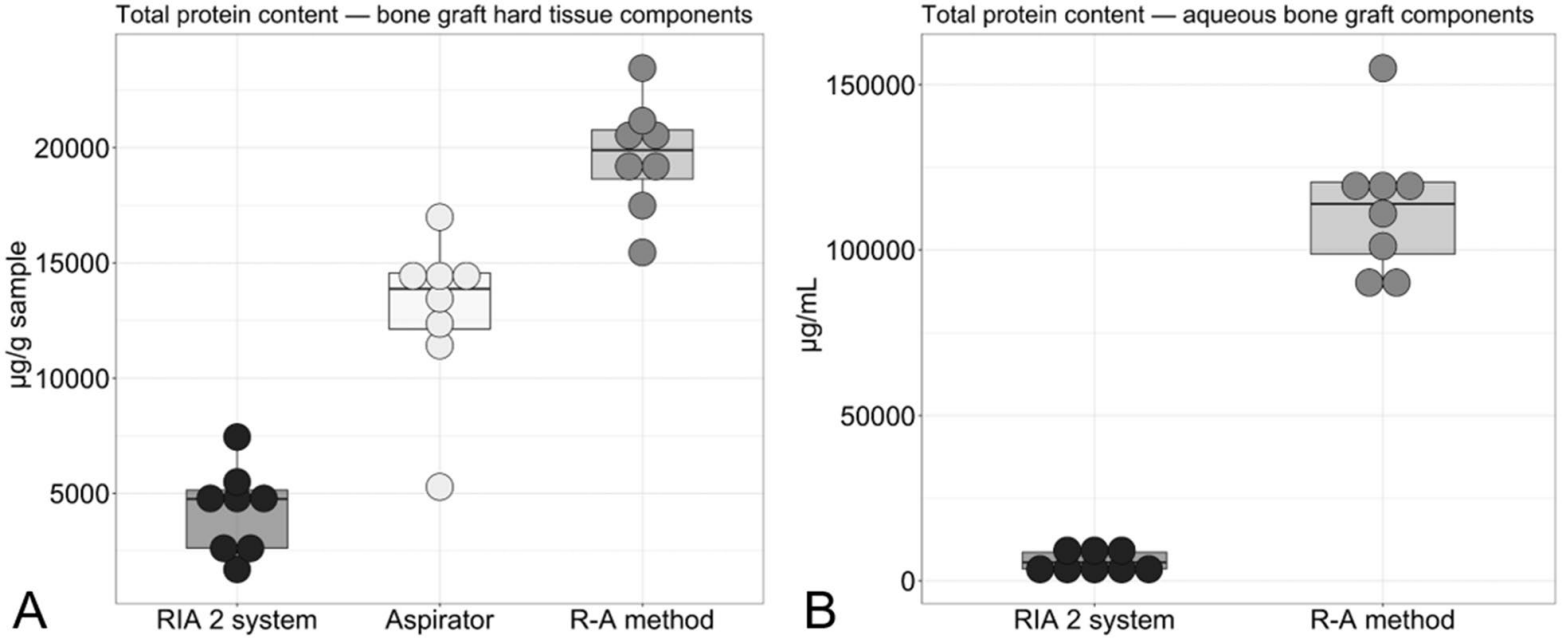
Boxplots of BG total protein amount by harvesting method. In the ARA group with BG harvested by either the aspirator prototype or the R–A method, there was a tendency for higher total protein in the BG hard tissue components **A** and the aqueous BG components **B** compared with the BG harvested by the RIA 2 system

**Fig. 6:**
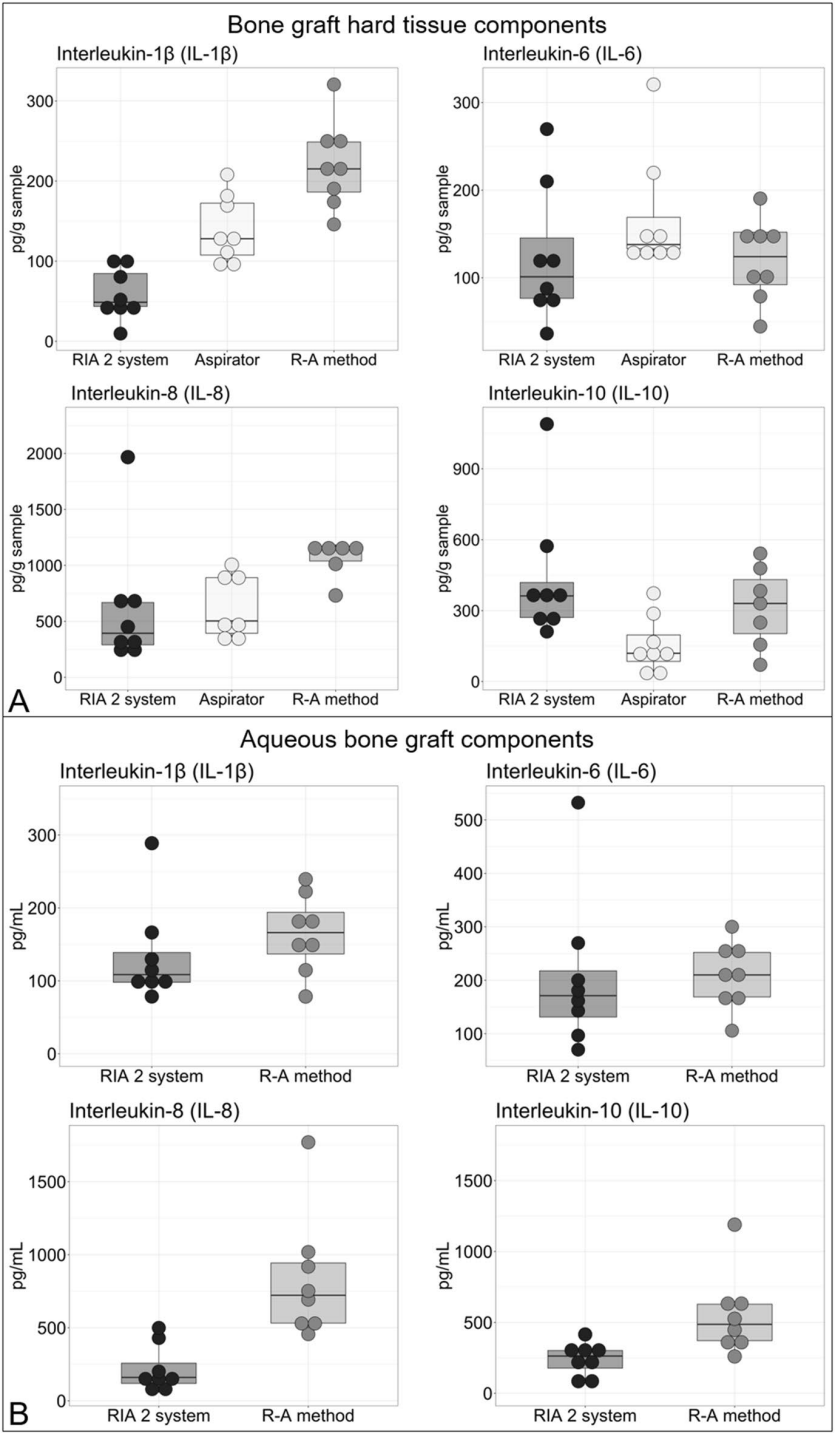
Boxplots of BG inflammatory cytokine amount by harvesting method. In the BG hard tissue components **A** and aqueous BG components **B**, a similar tendency toward higher inflammatory cytokine amounts was observed for the ARA group, which includes both the aspirator prototype and the R–A method, compared to the BG harvest obtained with the RIA 2 system. Please note that if less than eight dots per group are shown, missing measurements were above the detection range

GF analyses showed that the aspirator, alone, and in combination with sequential reaming and aspiration (R–A method), as well as the RIA 2 system, had the capacity to harvest BG and aqueous BG components rich in GFs. There were trends observable for some GFs measured with higher amount in the RIA 2 group (IGF-1, TGF-β1, FGF-2, BMP-4), whereas for VEGF and BMP-2, no such marked differences were observed (Fig. 4A). Except for VEGF and IGF-1, which were below detection range, all assessed GFs were detectable in the aqueous BG components (Fig. 4B). TGF-β1 and FGF-2 showed a trend toward higher content in the RIA 2 group aqueous BG components, whereas slightly more BMP-2 and BMP-4 were detected in the R–A method aqueous BG components. Total protein in the hard tissue component was lowest for the RIA 2 system, followed by BM harvested with the aspirator prototype and that harvested using the R–A method (Fig. 5A). Furthermore, in the aqueous BG component, the total protein content was higher when harvested with the R–A method compared to the RIA 2 system (Fig. 5B). The amount of pro-inflammatory cytokines IL-1β and IL-8 in BG hard tissue samples tended to be higher in the ARA group, whereas IL-6, as well as the anti-inflammatory cytokine IL-10, showed no such clear tendencies (Fig. 6A). For the IL-1β, IL-6, IL-8, and IL-10 content of the aqueous BG component, the trend was slightly higher for the harvest collected with R–A method compared to the RIA 2 system (Fig. 6B).

## Discussion

Transplantation of autologous BG remains a key procedure for orthopaedic surgeons, particularly since non-union is observed in 1.9–4.9% of all fracture cases and in 5.0–14.0% of cases following tibia fractures [69, 70]. Over the last 20 years, the RIA system has become an effective tool for harvesting femoral and tibial BG; however, to safely use this one-step aggressive reaming technique, a relevant learning curve must be followed, with training at “centers of excellence” recommended [71–73]. In addition, the financial constraints of this costly procedure must be considered [74]. More recently, regenerative medicine-based strategies with procedures using fresh BM have been developed [51]. Applying the RIA system does not allow for selectively harvesting BM and bone chips as well as relevant amounts of osteogenic and osteoinductive factors might be wasted in its filtrate (“waste-water”) [75–77]. To address these shortcomings, a highly versatile prototype of a novel BG collection aspirator that does not need additional use of irrigation fluid was developed. This allows for the initial selective harvesting of BM, followed by a two-step method with sequential reaming and aspiration to harvest BG (mainly consisting of bone chips) from the intramedullary canal.

Therefore, this study investigated the effectiveness of an original BG harvesting technology and the osteoinductive quality of the harvested BG. Comparisons were made with the latest version of the RIA system, termed the RIA 2 system. We have comprehensively and completely reported our experimental results and thus successfully averted publication bias, as well as selective analysis and outcome reporting bias [78]. Clearance of the medullary cavity from fatty BM along with reaming debris prior to intramedullary nailing was the primary indication and the initial incentive for the development of the RIA system [79]. However, since the RIA system can also be used to harvest considerable amounts of intramedullary BG [80, 81], novel devices with comparable application scope to be (preclinically and clinically) tested must compete especially in BG harvesting capacity. Sheep femur was used in this project as femur is the preferable harvesting site for intramedullary (endosteal) BG in human surgery. As the antegrade surgical approach to the sheep femur is challenging, particularly due to large soft tissue coverage [82], multiple cadaveric trials were performed prior to in vivo experiments, in order to mitigate potential risks of intraoperative complications.

After opening the proximal femur with standard reamers, we observed that the aspirator prototype was able to harvest BM without requiring additional irrigation fluids. About five times the amount of undiluted BM was aspirated from the medullary canal of a sheep femur compared to the maximum undiluted BM that can be aspirated from a human IC. Therefore, particularly, since the human femur is larger than the sheep femur [83], a small percutaneous access of 10 mm via the major trochanter may allow for collecting BM in large (undiluted) volumes. Interestingly, the use of clotted (unprocessed) BM, similar to our procedure, was recently deemed to have relevant biological effects, with in vitro higher growth kinetics of MSCs derived from clotted compared to unclotted BM [51], due to comprising degranulated platelets, which can deliver GFs and cytokines relevant to bone formation into the lesion site [84]. By applying this process, relevant factors can be released, including, but not limited to TGF-β1 and FGF, consistent with our findings [84, 85]. Consequently, in preclinical studies, the osteoinductive capacity of clotted BM has been tested in combination with cancellous bone matrix [85], osteogenic protein-1 (OP-1) [86], and porous β-tricalcium phosphate incorporated with gentamicin [87], all of which indicated relevant bone regenerative potential. The results of the corresponding clinical trials are currently underway [51]. Taken together, BM might play a key role in future regenerative treatment strategies, and for its collection, the novel aspirator prototype technology proved to have an ergonomic and intuitive design that allows effective harvesting of BM with high osteoinductive potency from the intramedullary canal of long bones.

For more than 30 years, human reaming debris has been well-known for its high osteogenic capacity, but standardized harvesting by extraction from the reamer head has been described as very tedious [1, 88]. In this study, both the RIA 2 and the ARA group yielded similar amounts (weight in grams) of BG, suggesting that the novel harvesting concept with its innovative aspirator prototype has comparable BG harvesting capacity. Importantly, analysis of pre- and postoperative CT scans of the femora showed that the reduction in cortical thickness was similar in both experimental groups. Due to the use of irrigation fluid under continuous potent intramedullary suction, complete extraction of BG is to be expected with the RIA 2 system. Therefore, since we observed a similar reduction in cortex volume in both groups, it is likely that very few, if any, morselized BG residuals remained in the medullary canal after application of the R–A method. This further supports the hypothesis that the R–A method is effective in harvesting BG.

Furthermore, the harvested BG was assessed for relevant signaling proteins for bone regeneration, including GFs and inflammatory cytokines. Different types of mechanical stimulation associated with BG harvesting, such as conventional reaming [44] and the RIA technique [46, 47], influence the quantity of GFs in harvested material; however, these techniques have not yet been directly compared. Giannoudis et al. [44] investigated the difference in GF quantities (PDGF, VEGF, IGF-1, TGF-β1, BMP-2) from femoral canal blood samples using ELISA before and after reaming and nail insertion in patients with femoral shaft fractures. Intramedullary reaming increased all studied GFs locally in the bone canal, except for BMP-2 levels, which were below detection range [44]. Using ELISA, Schmidmaier et al. [46] compared the quantities of human GFs (BMP-2, BMP-4, TGF-β1, IGF-1, FGF-1, FGF-2, PDGFbb, VEGF) derived from medullary reaming debris and the aspirated irrigation fluid of the RIA system to the BG obtained from the IC. Except for VEGF and FGF-2, they observed more GFs and a higher total protein in the RIA reaming debris compared to the harvested material of the IC (RIA graft 38.8 mg/g versus IC 18.3 mg/g) [46]. Thus, blood derived from the reaming debris had a higher GF content than BM [44], and reaming debris contained more GFs than the material harvested from the IC [46].

We observed for several GFs a tendency toward higher amounts for the RIA 2 group in solid BG components (IGF-1, TGF-beta1, FGF-2, and BMP-4) and in aqueous BG components (TGF-beta1 and FGF-2). Therefore, although relevant GF amounts were observed for both groups, our results did not entirely support our hypothesis. We hypothesized that diluting with saline, as with application of the RIA 2 system, would result in lower GF amount, compared to the novel R–A method. One explanation for the lower GF content in the R–A method compared to the RIA 2 system might be that the associated higher intramedullary peak temperatures of the conventional reaming might have led to thermal BG necrosis [89, 90], which in turn can either cause structural changes or even denaturation of proteins and, therefore, reduced detectability with ELISA. Whereas in the RIA 2 group, thermal osteonecrosis could be avoided by the room temperature irrigation solution and very sharp reamer blades used with the RIA system, which had been associated with decreased temperatures when compared with standard stepwise reaming [91]. It is well-recognized that the friction between instruments and bone and the local contact pressure are important factors in cortical heat generation [92]. Therefore, to minimize the risk of thermal BG necrosis due to friction between instruments and cortical bone and high local contact pressure, we used the latest generation intramedullary reaming system (i.e., Bixcut IM Reamer System; Stryker, Kalamazoo, MI), which has deeper flutes compared with standard AO/ASIF reamers (Synthes, Germany) exerting less friction and pressure as BG debris generated during reaming escapes more efficiently [93–95]. Yet, a possible negative effect on BG when using standard reamers due to heat generation during intramedullary reaming cannot be excluded and may be further investigated in future studies. Another explanation might be that a “matrix effect” occurred during the ELISA testing [96, 97]. During ELISA, all antigens being assayed are contained in a complex solution known as the “matrix” [98]. If the target analytes are not of high purity, ELISAs are sometimes susceptible to the matrix effect, in which an inaccurate result is obtained, because complete recovery of the analyte from the matrix sample is inhibited [96]. Since several proteins such as albumin and fibrinogen can cause interference with immunoassay (i.e., ELISA) measurements [97], the higher total protein content observed in the ARA group compared with the RIA 2 group, may be indicative of the presence of a matrix effect. Given that the ECM of the BGs in the ARA group was not exposed to the irrigation fluid, and because no anticoagulants were added to the BGs, this may indicate that more GFs in the ARA group were incorporated into a highly complex, protein-rich ECM compared with the RIA 2 group and, therefore, were not quantifiable by ELISA, which detects only soluble factors. Taken together, the results of this study indicate that both the BG hard tissue component and the aqueous BG component of the RIA 2 system, the aspirator prototype, and the R–A method contain relevant amounts of osteoinductive GFs. Moreover, as indicated in recent literature [99], our results suggest that the aqueous filtrates of the RIA system and, correspondingly, the undiluted aqueous BG components of the R–A method can increase the biological activity of the BG.

Moreover, we observed a trend toward higher amounts of several cytokines in BG harvested in the ARA group compared with the RIA 2 group. The origins and interactions between molecular factors, immune cells, bone macrophages, and progenitor cells are highly complex (Additional file 1: Fig. S6), and it is pivotal to recognize that these osteoimmunomodulatory factors initiate the (bone) repair/regeneration cascade by stimulating angiogenesis, attracting, and promoting differentiation of MSCs, and enhancing ECM synthesis [100–102]. Although immune cell composition and ensuing cytokine pattern are not completely understood yet [103], there is consistent evidence that bone regeneration is enhanced by promoting the acute inflammatory response with localized pro-inflammatory stimuli [104–107]. Immediately after injury, tissues physiologically exhibit higher levels of pro-inflammatory cytokines, such as IL-1β and IL-6, to facilitate early bone formation [102, 108]. A key factor might be that macrophage-derived cytokines regulate the formation and structure of blood clots [109]. Thus, the complex osteoimmunomodulative steps in the hematoma that surrounds the BG may be favorable or even essential for bone formation, as emphasized in recent studies observing that removal of early stage hematoma results in delayed bone healing or non-union [110, 111]. Moreover, a preserved blood clot surrounding the BG, as in the R–A method, can play a crucial role in bone regeneration by providing a fibrin scaffold that attracts MSCs to inwardly migrate, settle, and proliferate [110, 112]. However, before definitive conclusions can be drawn as to whether different amounts of pro- and anti-inflammatory cytokines in BG, collected using the new intramedullary harvesting technology including the R–A method, result in an increased capacity for bone regeneration, further in vivo studies with in-depth histological analyses are required.

### Limitations

Since a matrix effect during ELISA cannot be excluded, the protocol for BG digestion may be modified in subsequent studies to consider alternative methods of protein extraction and protease inhibitors (e.g., use of plasmin to break up the ECM) in addition to the use of collagenase. Furthermore, direct assessment of macrophage polarization rather than quantification of cytokines of BG harvest may be addressed in future studies, because it is relevant to assess prolonged or even chronic foreign body reaction elicited by macrophages [31], potentially resulting in graft integration failure [113]. Moreover, previous studies indicate that the timely termination of inflammation is essential for a regenerative healing process [114]. Therefore, the osteogenic capacity of the BG harvested with the novel aspirator prototype or the R–A method needs to be evaluated in controlled experimental in vivo bone formation studies in small and large animals, including interaction with biomaterials to assess the ‘‘double-edged sword’’ effect of specific inflammatory cytokines.

## Conclusion

The current study demonstrated that both the RIA 2 system and an alternative intramedullary BG harvesting concept using a novel aspirator device that does not require irrigation fluid can obtain high amounts of GF and pro- and anti-inflammatory cytokines in BG. Thus, based on the preclinical data presented, it can be hypothesized that the harvested BGs with the RIA 2 system, aspirator prototype and R–A method contain a complex environment of many growth and osteoimmunomodulatory factors that are able to provide the required physiological functions to achieve, facilitate, and accelerate bone tissue regeneration. However, further studies are needed to verify the promising findings of the original intramedullary harvesting technology with regard to in vivo osteogenicity of the harvest for bone regeneration.

### Supplementary Information

**Additional file 1:** Aspiration of aqueous components from bone grafts harvested using the RIA 2 system or the R–A method.

**Additional file 2: Table S1.** Recombinant and antibody pairing details and additional reagent details. **Fig. S1.** Depiction of representative preoperative measurement of intramedullary femoral canal size diameter using the open-source medical image viewer Horos (version 3.3.6). **Fig. S2.** Dissection of sheep femur and thigh region for in-depth understanding of surgical access to the proximal femur. **Fig. S3.** Instrument setup for surgical approach of the proximal left femur via the trochanteric fossa. **Fig. S4.** Sheep positioning and surgical approach to the left proximal femur. **Fig. S5.** Exemplary image of segmentation method for calculation of femoral cortical bone volume reduction. **Fig. S6.** Selection of essential signalling molecules for early bone healing and their sources from long bones.

## Data Availability

The data that support the findings of this study are available from the corresponding authors upon reasonable request.
