# Supplementary material for: An in vivo study to investigate an original intramedullary bone graft harvesting technology
Source: Eur J Med Res. 2023 Sep 15;28:349. doi: 10.1186/s40001-023-01328-8 (PMC10503043; doi:10.1186/s40001-023-01328-8)
Supplement: Supplementary file 2 — Additional file 2: Table S1. Recombinant and antibody pairing details and additional reagent details. Fig. S1. Depiction of representative preoperative measurement of intramedullary femoral canal size diameter using the open-source medical image viewer Horos (version 3.3.6). Fig. S2. Dissection of sheep femur and thigh region for in-depth understanding of surgical access to the proximal femur. Fig. S3. Instrument setup for surgical approach of the proximal left femur via the trochanteric fossa. Fig. S4. Sheep positioning and surgical approach to the left proximal femur. Fig. S5. Exemplary image of segmentation method for calculation of femoral cortical bone volume reduction. Fig. S6. Selection of essential signalling molecules for early bone healing and their sources from long bones. [file 40001_2023_1328_MOESM2_ESM.docx]

Additional file

Contents

[Supplementary table 2](#_Toc141279519)

[Suppl. Table 1. Recombinant and antibody pairing details and additional reagent details. 2](#_Toc141279520)

[Supplementary figures 3](#_Toc141279521)

[Suppl. Fig. 1. Depiction of representative preoperative measurement of intramedullary femoral canal size diameter using the open-source medical image viewer Horos (version 3.3.6). 3](#_Toc141279522)

[Suppl. Fig. 2. Dissection of sheep femur and thigh region for in-depth understanding of surgical access to the proximal femur. 4](#_Toc141279523)

[Suppl. Fig. 3. Instrument setup for surgical approach of the proximal left femur via the trochanteric fossa. 5](#_Toc141279524)

[Suppl. Fig. 4. Sheep positioning and surgical approach to the left proximal femur. 6](#_Toc141279525)

[Suppl. Fig. 5. Exemplary image of segmentation method for calculation of femoral cortical bone volume reduction. 7](#_Toc141279526)

[Suppl. Fig. 6. Selection of essential signalling molecules for early bone healing and their sources from long bones (adapted from Ref. [2]). Created with BioRender.com. 8](#_Toc141279527)

[References 10](#_Toc141279528)

Additional file table

Additional file 2: Table S1. Recombinant and antibody pairing details and additional reagent details.

|  | **IL-1β** | **IL-6** | | **IL-8** | | **IL-10** |
| --- | --- | --- | --- | --- | --- | --- |
| **Recombinant protein** | Ovine IL-1β - 5 μg | Ovine IL-6 - 5 μg | | Ovine IL-8 - 5 μg | | Bovine IL-10 - 5 μg |
|  | Cat# RP0656V-005 | Cat# RP0367V-005 | | Cat# RP0488V-005 | | Cat# RP0379B-005 |
|  | (Kingfisher BioTech, MN, USA) | (Kingfisher BioTech, MN, USA) | | (Kingfisher BioTech, MN, USA) | | (Kingfisher BioTech, MN, USA) |
| **Capture antibody** | Monoclonal mouse anti-sheep IL-1β - 1 mg/mL | Monoclonal mouse anti-sheep IL-6 - 1 mg/mL | | Monoclonal mouse anti-sheep IL-8 - 1 mg/mL | | Monoclonal mouse anti-bovine IL-10 - 1 mg/mL |
|  | Clone 1D4 | Clone 4B6 | | Clone 8M6 | | Clone CC318 |
|  | Cat# MCA1658 | Cat# MCA1659 | | Cat# MCA1660 | | Cat# MCA2110 |
|  | (Bio-Rad, CA, USA) | (Bio-Rad, CA, USA) | | (Bio-Rad, CA, USA) | | (Bio-Rad, CA, USA) |
| **Detection antibody** | Polyclonal rabbit anti-sheep IL-1β - 0.1 mL | Polyclonal rabbit anti-sheep IL-6 - 0.1 mL | | Polyclonal rabbit anti-sheep IL-8 - 0.1 mL | | Monoclonal mouse anti-bovine biotin - 1 mg/mL |
|  | Cat# AHP423 | Cat# AHP424 | | Cat# AHP425 | | Cat# MCA2111B |
|  | (Bio-Rad, CA, USA) | (Bio-Rad, CA, USA) | | (Bio-Rad, CA, USA) | | (Bio-Rad, CA, USA) |
| **Secondary antibody** | Polyclonal goat anti-Rabbit IgG HRP - 1 mg/mL | Polyclonal goat anti-Rabbit IgG HRP - 1 mg/mL | | Polyclonal goat anti-Rabbit IgG HRP - 1 mg/mL | | ExtrAvidin HRP |
|  | Cat# A16110 | Cat# A16110 | | Cat# A16110 | | Cat# E2886 |
|  | (Thermo Fisher Scientific, MA, USA) | (Thermo Fisher Scientific, MA, USA) | | (Thermo Fisher Scientific, MA, USA) | | (Sigma-Aldrich, MO, USA) |
| **Reagent/item** | | | **Cat#** | | **Supplier** | |
| Carbonate-bicarbonate buffer | | | C3041-50CAP | | Sigma-Aldrich, MO, USA | |
| 3,3′,5,5′-Tetramethylbenzidine (TMB) Liquid Substrate System | | | T8665-100ML | | Sigma-Aldrich, MO, USA | |
| Sulfuric acid (H_2_SO_4_) | | | 258105-100ML | | Sigma-Aldrich, MO, USA | |
| Nunc-Immuno™ MicroWell™ 96 well solid plates | | | M9410-1CS | | Sigma-Aldrich, MO, USA | |
| IL = interleukin; Cat# = catalogue number. | | | | | | |

## Additional file

## Additional file 2. Fig. S1. Depiction of representative preoperative measurement of intramedullary femoral canal size diameter using the open-source medical image viewer Horos (version 3.3.6).


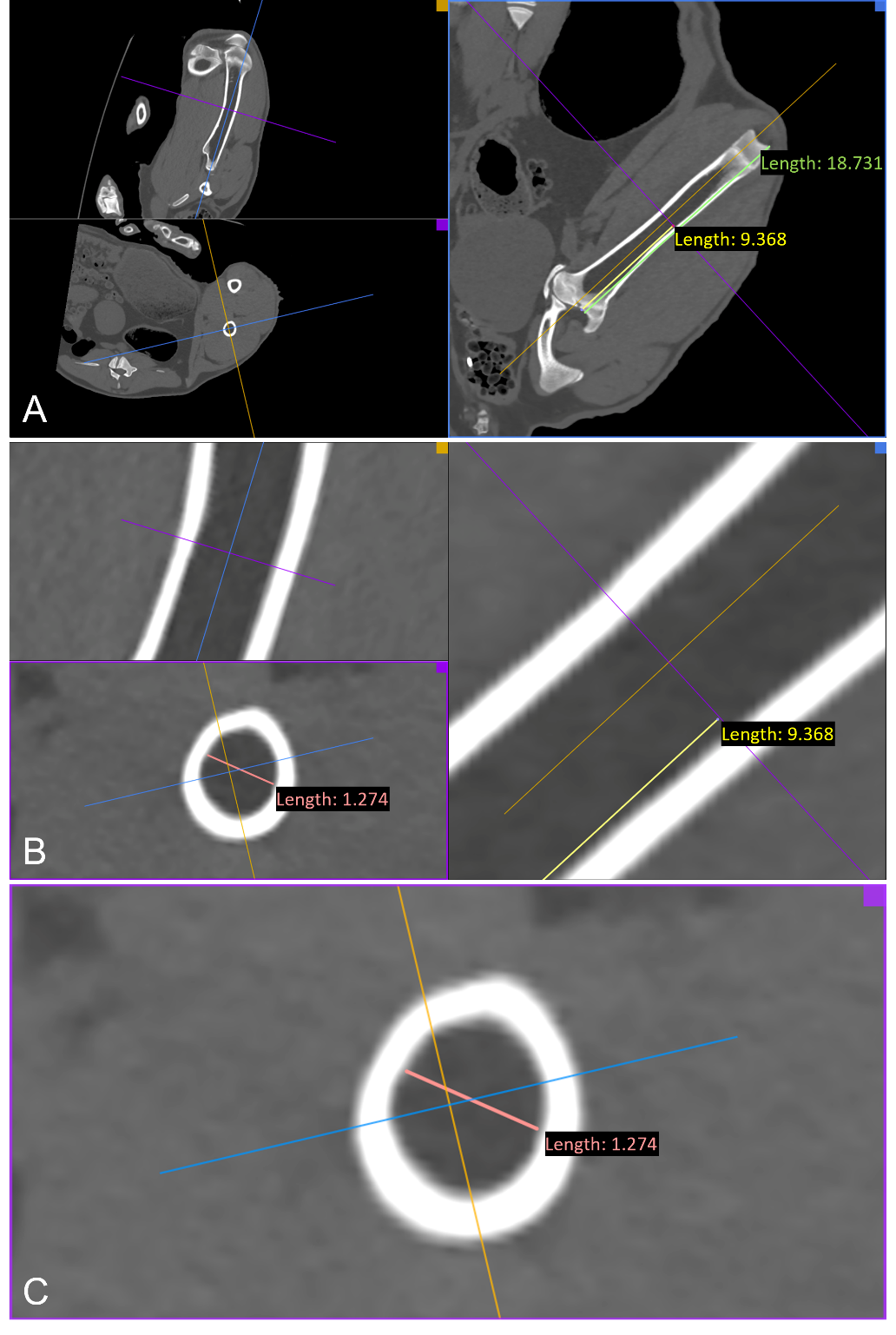


A: Using three computed tomography imaging planes, a line parallel to the cortex was drawn from the trochanteric fossa to the condyle (green line). In the middle of this line (indicated with a yellow line), another line was drawn perpendicular to it (purple line). B: The area of the perpendicular line was enlarged to confirm the correct position. C: Finally, the smallest diameter was calculated by manually drawing a line at the narrowest point of the intramedullary canal (pink line).

## Additional file 2. Fig. S2. Dissection of sheep femur and thigh region for in-depth understanding of surgical access to the proximal femur.


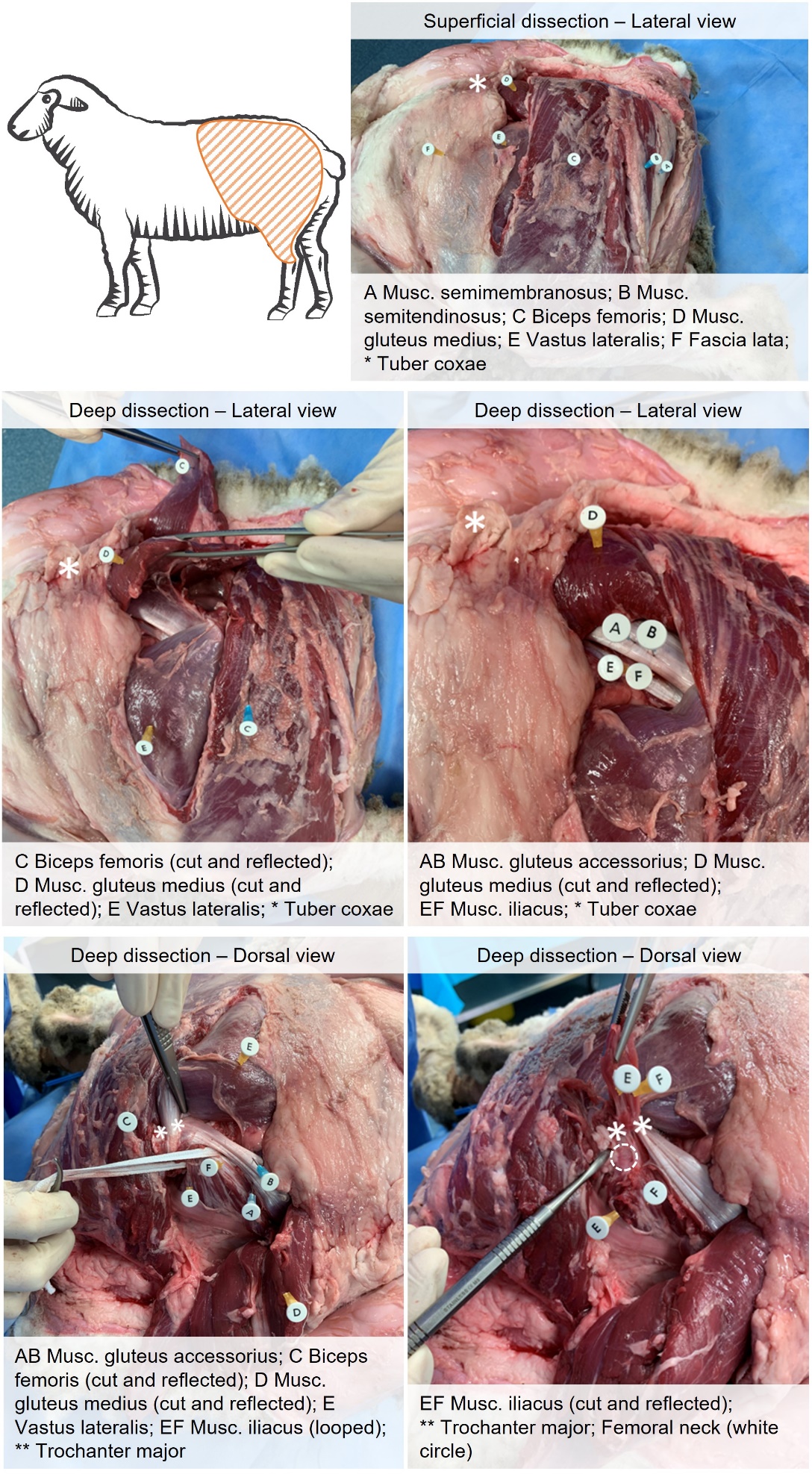


## Additional file 2. Fig. S3. Instrument setup for surgical approach of the proximal left femur via the trochanteric fossa.


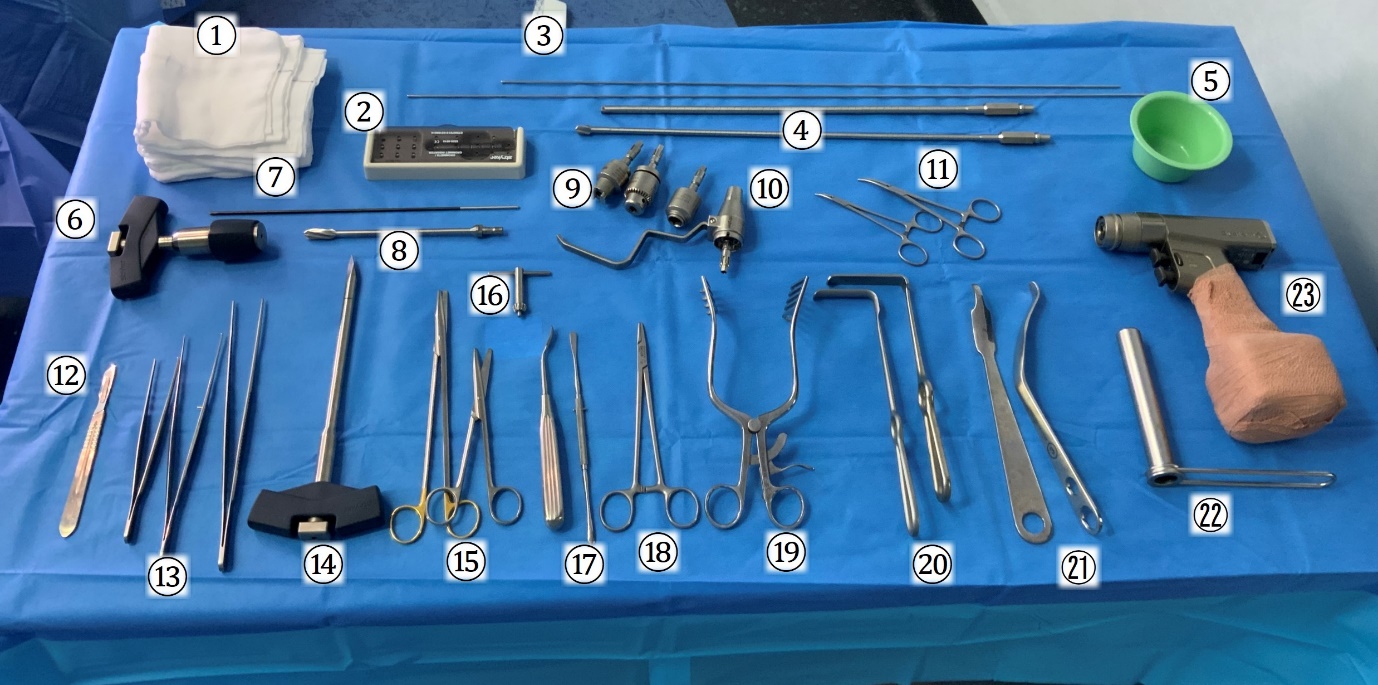


Key: 1 = Abdominal sponges; 2 = Grommet inserter/extractor and grommets; 3 = Ball tip guide wire; 4 = Bixcut fixed-head system Ø11.0 mm; 5 = Small plastic bowl; 6 = Guide wire T-handle with chuck; 7 = K-wire 3 × 285 mm; 8 = Rigid reamer Ø10 mm; 9 = Lockable chucks (Stryker 1/4" Jacobs chuck, Stryker Hudson/Modified Trinkle drill, Stryker Large/ AO Reamer); 10 = Stryker System 6 drill K-wire attachment; 11 = Artery forceps; 12 = Scalpel blade holder with Swann Morton scalpel blade No 10; 13 = Surgical and anatomical forceps; 14 = Straight awl Ø10 mm; 15 = Metzenbaum dissection scissor with curved tip and dressing scissor; 16 = Key for drill chuck; 17 = Periosteal elevators; 18 = Large needle holder; 19 = Large self-retaining retractor; 20 = Langenbeck retractors medium and large size; 21 = Hohmann retractor and modified blunt Hohmann retractor; 22 = Tissue protector; 23 = Stryker System 6 orthopaedic power tool with battery wrapped in sterile Coban wrap

## Additional file 2. Fig. S4. Sheep positioning and surgical approach to the left proximal femur.


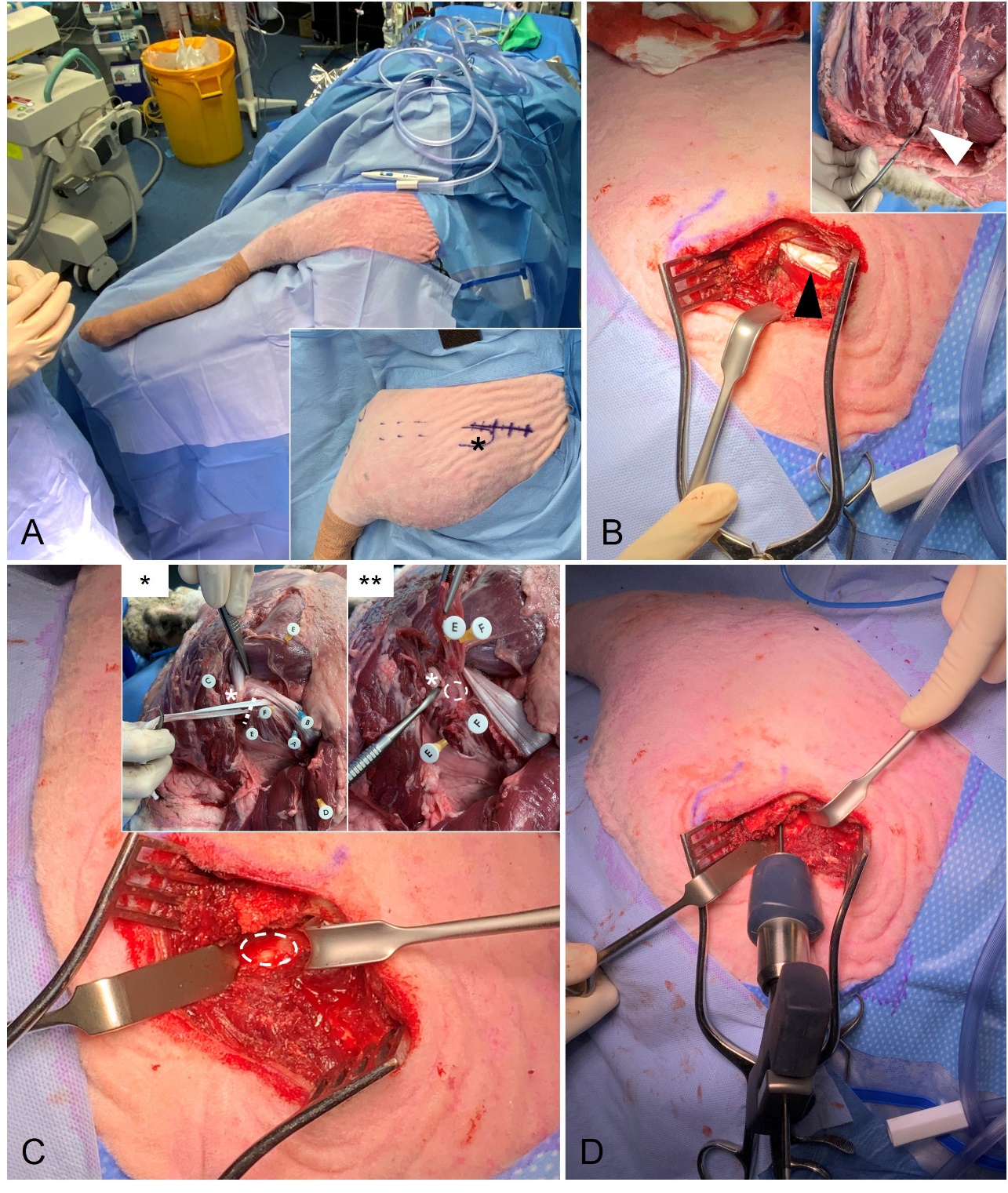


A: Sheep in right lateral recumbency under aseptic surgical condition to allow harvesting of graft material suitable for further processing. The inset in A is showing dashed lines from the trochanter major (black asterisk) to the patellar ligament to indicate the underlying femur. Note incision marking medial to the trochanter major. B: After incision of skin and musc. biceps femoris (white triangle in inset) the musc. iliacus is identified (black triangle). C: A Langenbeck retractor is placed next to the trochanter major (indicated white asterisk in both insets) and another Langenbeck retractor close to the femoral head. The musc. iliacus (labelled as EF) is cut following the dashed line as shown in the first inset (*) for access to the trochanteric fossa via the femoral neck (dashed white circle). D: To guide the Ø10 mm rigid reamer a K-wire is manually introduced in the intramedullary canal via the trochanteric fossa using a T-handle equipped with a chuck.

## Additional file 2. Fig. 5. Exemplary image of segmentation method for calculation of femoral cortical bone volume reduction.


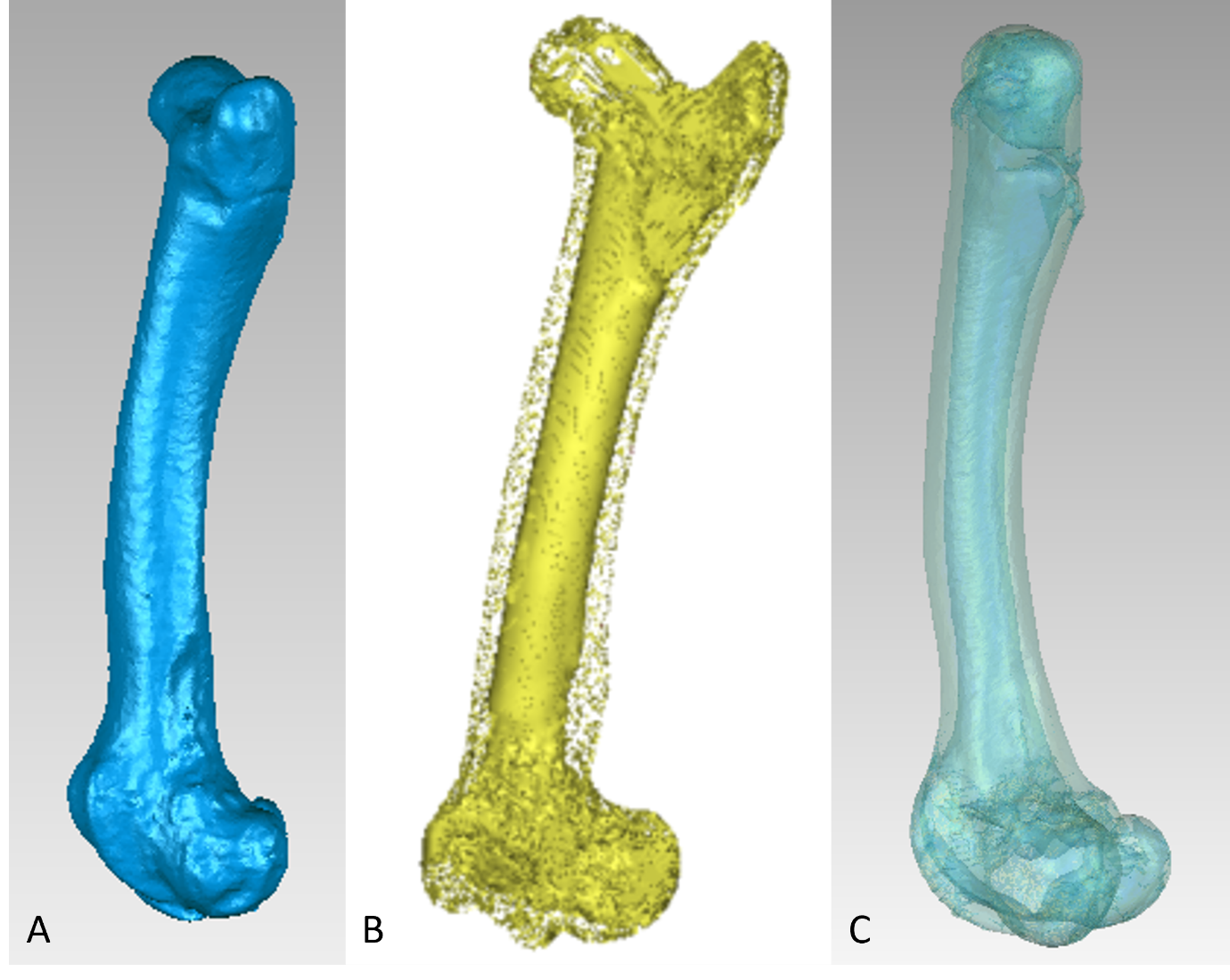


The protocol was applied to the preoperative as well as postoperative CT scans and difference of cortical volume represents the cortical reduction after reaming of the intramedullary canal. A: First, to derive the cortical shape an upper threshold of 1200 Hounsfield Units (HU) was selected. B: Similar to the validated protocol of Aamodt et al. [1], a threshold of 580 HU was selected to determine the interface between spongious and cortical bone to depict the intramedullary canal. C: In a last step, the reduction of cortical volume is derived by subtraction of spongy shape from cortical shape.

## Additional file 2. Fig. S6. Selection of essential signalling molecules for early bone healing and their sources from long bones (adapted from Ref. [2]). Created with BioRender.com.


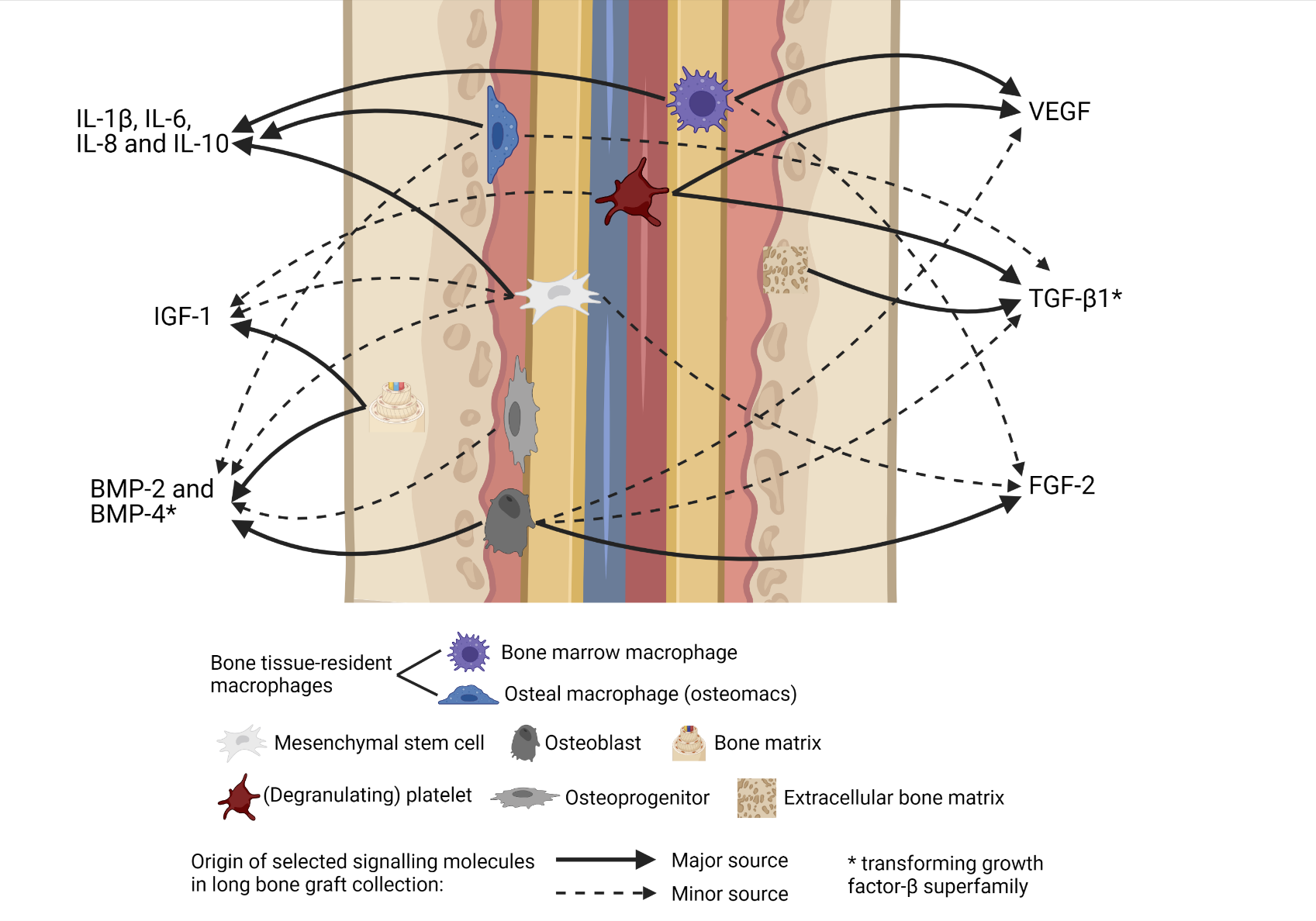


In addition to structural proteins, long bones contain very potent regulators of bone cell metabolism such as non-collagen proteins of growth factors (GFs) and inflammatory cytokines.

Bone GFs are produced by osteoblasts and incorporated into the extracellular matrix during bone formation but can also be taken up systemically from the serum in small quantities and incorporated into the matrix. The GFs remain in the matrix until remodelling or trauma causes solubilisation and release of the proteins [3, 4]. Once released, the GFs are able to regulate osteoblast and osteoclast metabolism during bone remodelling and initiate and control a healing response after bone trauma. The exact source of the various GFs in long bone is very complex and not yet fully known. In brief, insulin-like growth factor 1 (IGF-1) is mainly released by mature bone matrix [5], platelets [6], and mesenchymal stem cells (MSC) in granulation stage [7]. Bone morphogenetic protein (BMP)-2 and BMP-4 are derived from osteoprogenitors and MSCs, platelets, osteoblasts, bone extracellular matrix and mature bone matrix as well as chondrocytes [7-9]. After their release, the extracellular matrix functions as a temporary storage for BMPs. BMP-2 appears to be strongly induced immediately after injury during the period when mesenchymal cells are being recruited to the site of injury and chondrogenesis is being promoted [10]. Transforming growth factor (TGF)-ß1 is mainly derived from osteoblasts [11], degranulating platelets [6, 12] and the extracellular matrix [7, 12, 13]. TGF-β1 is a cytokine present in the bone matrix, residing in its inactive form by remaining non-covalently bound to a latency-associated protein (LAP) until cleaved and activated by osteoclasts [14]. TGF- β1 and bone morphogenetic proteins such as BMP-2 and BMP-4 belong to the (TGF)-β superfamily. Noteworthy, the bone-inducing BMPs can be divided into several subgroups, according to homology of their amino acid sequences [15, 16] with BMP-2 and BMP-4 comprising one osteogenic subgroup (subfamily 1) [17].

Particularly platelets form a rich source of important growth factors [18]. Alpha granules of platelets contain biologically active proteins such as TGF-β, fibroblast growth factor (FGF), IGF-1, and vascular endothelial growth factor (VEGF) [6, 19]. When integrity of vascularized osseous tissue is manually disturbed hematoma consisting of red blood cells and neutrophils is formed at the injury site. For instance, VEGF is then highly concentrated in the hematoma after bone injury as hypoxia in hematoma induces VEGF expression, and the fibrin matrix in hematoma may serve as a reservoir of VEGF [20]. Moreover, hypoxia in the hematoma induces VEGF expression in surrounding bone cells or recruited inflammatory cells. Subsequently, VEGF released from fragmented bone matrix, platelets or infiltrated innate immune cells may bind to the heparin associated with fibrin(nogen), and be sequestrated in the fibrin matrix, turning fibrin matrix in the hematoma into a reservoir of VEGF [6, 21-24]. In addition, osteoblasts are also a major source of angiogenic factors, including VEGF and thereby stimulate angiogenesis [20, 25]. Further, ‘basic’ FGF (FGF-2) is mainly derived from macrophages, MSCs and osteoblasts [7]. Interestingly, it has been observed that FGF-2 is generally more potent than ‘acidic’ FGF (FGF-1) [26]. The inflammatory cytokines interleukin (IL)-1β, IL-6, IL-8, and IL-10 are mainly released by MSC and macrophages [11] including bone tissue-resident macrophages [27, 28].

# References

1. Aamodt A, Kvistad KA, Andersen E, Lund-Larsen J, Eine J, Benum P, et al. Determination of Hounsfield value for CT-based design of custom femoral stems. The Journal of bone and joint surgery British volume. 1999;81(1):143-7.

2. Rijal G, Li W. Native-mimicking in vitro microenvironment: An elusive and seductive future for tumor modeling and tissue engineering. Journal of Biological Engineering. 2018;12.

3. Canalis E, McCarthy T, Centrella M. Growth factors and the regulation of bone remodeling. The Journal of clinical investigation. 1988;81(2):277-81.

4. Joyce ME, Jingushi S, Bolander ME. Transforming growth factor-beta in the regulation of fracture repair. The Orthopedic clinics of North America. 1990;21(1):199-209.

5. Seck T, Scheppach B, Scharla S, Diel I, Blum WF, Bismar H, et al. Concentration of insulin-like growth factor (IGF)-I and -II in iliac crest bone matrix from pre- and postmenopausal women: relationship to age, menopause, bone turnover, bone volume, and circulating IGFs. J Clin Endocrinol Metab. 1998;83(7):2331-7.

6. Nurden AT, Nurden P. Platelets at the Interface between Inflammation and Tissue Repair. In: Anitua E, Cugat R, Sánchez M, editors. Platelet Rich Plasma in Orthopaedics and Sports Medicine. Cham: Springer International Publishing; 2018. p. 13-27.

7. Dimitriou R, Tsiridis E, Giannoudis PV. Current concepts of molecular aspects of bone healing. Injury. 2005;36(12):1392-404.

8. Sipe JB, Zhang J, Waits C, Skikne B, Garimella R, Anderson HC. Localization of bone morphogenetic proteins (BMPs)-2, -4, and -6 within megakaryocytes and platelets. Bone. 2004;35(6):1316-22.

9. Pecina M, Vukicevic S. Biological aspects of bone, cartilage and tendon regeneration. Int Orthop. 2007;31(6):719-20.

10. Cho TJ, Gerstenfeld LC, Einhorn TA. Differential temporal expression of members of the transforming growth factor beta superfamily during murine fracture healing. J Bone Miner Res. 2002;17(3):513-20.

11. Upadhyaya V, Arora A, Goyal A. Bioactive Platelet Aggregates: Prp, Prgf, Prf, Cgf And Sticky Bone. IOSR Journal of Dental and Medical Sciences. 2017;16:05-11.

12. Lind M. Growth factor stimulation of bone healing. Acta orthopaedica Scandinavica. 1998;69(sup283):i-37.

13. Lieberman JR, Daluiski A, Einhorn TA. The Role of Growth Factors in the Repair of Bone : Biology and Clinical Applications. JBJS. 2002;84(6):1032-44.

14. Perez JR, Kouroupis D, Li DJ, Best TM, Kaplan L, Correa D. Tissue Engineering and Cell-Based Therapies for Fractures and Bone Defects. Frontiers in Bioengineering and Biotechnology. 2018;6.

15. Tsiridis E, Upadhyay N, Giannoudis P. Molecular aspects of fracture healing: which are the important molecules? Injury. 2007;38 Suppl 1:S11-25.

16. Li JZ, Li H, Sasaki T, Holman D, Beres B, Dumont RJ, et al. Osteogenic potential of five different recombinant human bone morphogenetic protein adenoviral vectors in the rat. Gene Ther. 2003;10(20):1735-43.

17. Miyazono K, Kamiya Y, Morikawa M. Bone morphogenetic protein receptors and signal transduction. J Biochem. 2010;147(1):35-51.

18. Nikolidakis D, Jansen JA. The biology of platelet-rich plasma and its application in oral surgery: literature review. Tissue engineering Part B, Reviews. 2008;14(3):249-58.

19. Blair P, Flaumenhaft R. Platelet alpha-granules: basic biology and clinical correlates. Blood Rev. 2009;23(4):177-89.

20. Hu K, Olsen BR. The roles of vascular endothelial growth factor in bone repair and regeneration. Bone. 2016;91:30-8.

21. Street J, Winter D, Wang JH, Wakai A, McGuinness A, Redmond HP. Is Human Fracture Hematoma Inherently Angiogenic? Clinical Orthopaedics and Related Research®. 2000;378:224-37.

22. Martino MM, Briquez PS, Ranga A, Lutolf MP, Hubbell JA. Heparin-binding domain of fibrin(ogen) binds growth factors and promotes tissue repair when incorporated within a synthetic matrix. Proc Natl Acad Sci U S A. 2013;110(12):4563-8.

23. Mosesson MW. Fibrinogen and fibrin structure and functions. J Thromb Haemost. 2005;3(8):1894-904.

24. Oryan A, Alidadi S, Moshiri A. Platelet-rich plasma for bone healing and regeneration. Expert Opinion on Biological Therapy. 2016;16(2):213-32.

25. Wang Y, Wan C, Deng L, Liu X, Cao X, Gilbert SR, et al. The hypoxia-inducible factor alpha pathway couples angiogenesis to osteogenesis during skeletal development. The Journal of clinical investigation. 2007;117(6):1616-26.

26. Canalis E, McCarthy TL, Centrella M. Growth factors and cytokines in bone cell metabolism. Annu Rev Med. 1991;42:17-24.

27. Akdis M, Aab A, Altunbulakli C, Azkur K, Costa RA, Crameri R, et al. Interleukins (from IL-1 to IL-38), interferons, transforming growth factor β, and TNF-α: Receptors, functions, and roles in diseases. Journal of Allergy and Clinical Immunology. 2016;138(4):984-1010.

28. Harmer D, Falank C, Reagan MR. Interleukin-6 Interweaves the Bone Marrow Microenvironment, Bone Loss, and Multiple Myeloma. Front Endocrinol (Lausanne). 2018;9:788.
